# Obituary for Prof. Dr. Ulrich Siebenlist

**DOI:** 10.3390/biomedicines9030244

**Published:** 2021-03-01

**Authors:** Philip M. Murphy, Michael J. Lenardo

**Affiliations:** Laboratory of Molecular Immunology, NIAID, NIH, Bethesda, MD 20892, USA; mlenardo@niaid.nih.gov

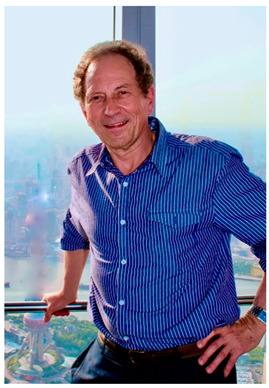



Ulrich Siebenlist, Ph. D., 1951–2020.

Ulrich “Uli” Siebenlist, our cherished colleague, passed away on 4 August 2020 after a long struggle with pancreatic cancer. Uli was a brilliant and pioneering molecular immunologist who did foundational work in immune system signal transduction. He spent his entire independent research career beginning in 1984 in the Division of Intramural Research of the National Institute of Allergy and Infectious Diseases (NIAID) at the National Institutes of Health (NIH) campus in Bethesda, Maryland. He was also a profile in courage until his death, as he nurtured his research to publication and mentored his staff toward their career goals.

Uli was born in Giessen, Germany but emigrated to Arizona with his family as a teenager. He obtained his undergraduate degree in physics at the University of Arizona, then earned a Ph.D. in Biophysics in 1979 from Harvard working with Nobel laureate Walter Gilbert. It was there that he developed a lifelong interest in the molecular basis of gene regulation, publishing seminal papers in *Nature* and *Cell* that elucidated the structure and function of the bacteriophage T7 promoter.

For his postdoctoral training, Uli turned to eukaryote gene regulation and one of the major problems of that era—immunoglobulin gene regulation, briefly moving to Bethesda and Lasker laureate Philip Leder’s Laboratory of Molecular Genetics at the National Institute of Child Health and Human Development (NICHD), NIH. He then moved back to Harvard when Leder became the head of the Department of Genetics at Harvard Medical School. Uli again made major discoveries at that time, publishing seminal papers in *Nature* and *Cell* on the structural organization of the IgD and c-myc loci.

From Harvard, Uli was recruited back to the NIH intramural program by Tony Fauci as an investigator in the Laboratory of Immunoregulation, NIAID, to apply molecular approaches to the problem of immune activation, eventually becoming the founding Chief of the Immune Activation Section. At NIH, Uli conducted powerful differential gene screens that advanced the field, mining important genes from mitogen-activated T cells, including those encoding two of the first known chemokines (CCL3 and CCL4, *Journal of Immunology* 1989), the major NF-κB transcription factor family members p50/NFκB1 (*Nature* 1990), p52/NFκB2 (*Molecular and Cell Biology* 1992) and RelB (*Oncogene* 1994), and the IL-17 signal transducer and NF-κB regulator CIKS (cytokine inducer of kappa B signaling, also known as Act-1; *PNAS* 2000).

Building on these foundational genetic discoveries, Uli’s lab went on to study how NF-κB is regulated at the biochemical level and, at the biological level, how NF-κB governs immune responses. His lab’s 1995 *Science* paper identified specific amino acid positions in the NF-κB inhibitor IκB-α, which become phosphorylated upon T-cell activation. This is a critical step that is needed for IκB- α degradation, release of NF-κB inhibition, and subsequent translocation of NF-κB to the nucleus to activate target genes. Their 1993 *Genes and Development* paper reported the construction of p50 and p52 single- and double-knockout mice, which revealed molecular redundancy as a fundamental feature of the system with impaired immune system development, including defective osteoclast development resulting in osteopetrosis, only occurring when both components were missing.

The lab’s 2000 *PNAS* paper identified CIKS as an upstream adaptor linking T-cell activation signals to NF-κB activation, by binding to NEMO/IKKγ. Later in 2009 in *Immunity*, Uli and his team provided evidence that CIKS acts as a promiscuous adaptor for signaling by IL-17 family members, through their surface receptors, and identified a major role for CIKS in allergic airway inflammation.

Uli reserved special devotion and 27 papers to understanding the atypical multitasking IκB family member Bcl-3. The lab’s 1992 *Nature* paper showed that Bcl-3 can help induce some NF-κB-regulated genes, by antagonizing inhibitory effects of p50 homodimers. The next year in *Cell*, they described how Bcl-3 uniquely resides constitutively in the nucleus, where it can directly transactivate genes through kappa B motifs, by associating preferentially with DNA-binding p50 homodimers. In a series of *Immunity* papers published in 1997, 2007, and 2014, they showed that Bcl-3 regulates diverse immunologic processes, including T cell-mediated immunity, splenic development, the germinal center reaction, the plasticity, and pathogenicity of autoimmune T-cells, and central immunologic tolerance.

Uli moved his lab to the Laboratory of Molecular Immunology of NIAID in 2012 and continued to publish important papers on NF-κB signaling but gravitated towards disease contexts, including autoimmunity, allergy, infectious disease, and cancer. In his latest papers, which were published posthumously in *PLoS Pathogens* and *Immunology and Cell Biology*, Uli further delineated cell intrinsic and cell autonomous roles of Bcl-3 in the differentiation of regulatory T cells and LCMV-specific CD8^+^ memory T cells.

This is only a small sampling of Uli’s profound impact on immunology. One global measure is his H-factor of 69; another is that 10% of his scholarly output was published in *Science*, *Nature*, *Cell*, and *Immunity*. His 1994 *Annual Review of Cell Biology* synthesis of the NF-κB field is a classic with 2724 citations and counting. He was also a past Chair of an NF-κB Keystone Conference.

Uli was a scientist’s scientist, passionate about his work, with a vision for the big picture but rigorous to the bone for the details. Every paper was a complete and elegant work typically bearing the fruit of direct tests of hypotheses, integrated genetic and biochemical analyses, the latest cutting-edge technologies, and complex genetic mouse models. All of which he created to enable precise, cell-type-specific statements about the function of genes confusingly scattered across the many cellular outposts of the immune system. He relished the challenges of understanding the NF-κB system from extracellular stimulus to the genome.

Uli made everyone around him a better scientist. He possessed an innate humility and gently gave generous and constructive criticism that went to the heart of an issue and helped advance the science. He blended the quantitative rigor of a physicist with the tolerance of a biologist for complexity, ambiguity, and context, all shaped by an expansive curiosity. He was a staunch proponent of the central importance of basic science but kept an eye out for clinical relevance. He freely shared his ideas and unique experimental tools. He cared profoundly about discovery and science and truth in general, and did not at all care about promoting himself. Like the athlete and coach he was at home, he did not shrink from competition in the lab, but he competed fairly and did not cut corners to steal victory for himself. Uli was in it for truth, not glory, and his discoveries stood the test of time.

Uli also made everyone around him a better human being. He had great integrity and relentless loyalty to all his staff. He had a finely tuned sense of irony and an eye for the absurd, which made conversation with him endlessly lively, insightful, and fun. He loved the outdoors, played, and coached soccer, knew details about different sports and sport teams, especially his beloved Washington Nationals (champions in 2019, at last). He also seemed to know everything that was going on at NIH. He lived life to the fullest and would often return to his beloved Western United States, to recharge his batteries.

Uli talked frankly about his disease, and in typical fashion quickly became well-versed in the basic science and clinical state-of-the-art in pancreatic cancer, and participated in a clinical trial. He decided early on not to retire, saying he wanted to help his lab family fulfill their professional goals as long as he could. Watching Uli relentlessly pursue this and succeed, publishing five papers in his final year, with others still to come posthumously, while enduring one of the most extraordinary challenges life offers, was for us a front row seat in watching a courageous and devoted scientist that we will never forget.

In the annals of immunology, Uli Siebenlist will be remembered as a paragon of excellence and an architect of immune cell signaling paradigms. In the hearts of his colleagues, he will be remembered as an inspirational and cherished colleague and friend. In addition to the Immunology community at NIH and beyond that he helped shape, Uli leaves behind his beloved family, including his wife and frequent coauthor, Kathy Kelly of the National Cancer Institute, NIH, and their two sons, Nicholas and Patrick, with sorrow for our shared loss, but vivid memories and gratitude for Uli’s time with us.

